# Double Digest RADseq: An Inexpensive Method for *De Novo* SNP Discovery and Genotyping in Model and Non-Model Species

**DOI:** 10.1371/journal.pone.0037135

**Published:** 2012-05-31

**Authors:** Brant K. Peterson, Jesse N. Weber, Emily H. Kay, Heidi S. Fisher, Hopi E. Hoekstra

**Affiliations:** Department of Organismic & Evolutionary Biology, Department of Molecular & Cellular Biology, Museum of Comparative Zoology, Harvard University, Cambridge, Massachusetts, United States of America; Natural History Museum of Denmark, University of Copenhagen, Denmark

## Abstract

The ability to efficiently and accurately determine genotypes is a keystone technology in modern genetics, crucial to studies ranging from clinical diagnostics, to genotype-phenotype association, to reconstruction of ancestry and the detection of selection. To date, high capacity, low cost genotyping has been largely achieved via “SNP chip” microarray-based platforms which require substantial prior knowledge of both genome sequence and variability, and once designed are suitable only for those targeted variable nucleotide sites. This method introduces substantial ascertainment bias and inherently precludes detection of rare or population-specific variants, a major source of information for both population history and genotype-phenotype association. Recent developments in reduced-representation genome sequencing experiments on massively parallel sequencers (commonly referred to as RAD-tag or RADseq) have brought direct sequencing to the problem of population genotyping, but increased cost and procedural and analytical complexity have limited their widespread adoption. Here, we describe a complete laboratory protocol, including a custom combinatorial indexing method, and accompanying software tools to facilitate genotyping across large numbers (hundreds or more) of individuals for a range of markers (hundreds to hundreds of thousands). Our method requires no prior genomic knowledge and achieves per-site and per-individual costs below that of current SNP chip technology, while requiring similar hands-on time investment, comparable amounts of input DNA, and downstream analysis times on the order of hours. Finally, we provide empirical results from the application of this method to both genotyping in a laboratory cross and in wild populations. Because of its flexibility, this modified RADseq approach promises to be applicable to a diversity of biological questions in a wide range of organisms.

## Introduction

**Figure 1:**
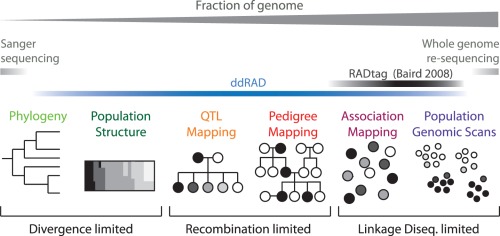
A flexible genotyping method can be used to optimize the number of genetic markers for a specific experimental approach in a given biological system. Segregating genetic markers are used to make inferences about historical processes (e.g., phylogenetic relationships, population structure) and functional mechanisms (e.g., genotype-phenotype mapping), but the optimal number of markers (fraction of the genome) needed to achieve a desired level of resolution differs based on both the experimental approach and the specific biological system–the number of genetic markers needed to recover relationships among populations or species is related to divergence among groups (e.g., more recent or more rapid events require more variable loci); the number of markers required for optimal resolution in phenotype-mapping experiments (conducted in laboratory crosses or pedigreed wild populations) is a function of the number of recombination events captured in the pedigree; the number of markers used in association mapping or selection scans in wild populations is determined by genome-wide levels of linkage disequilibrium, which is largely dictated by demographic history. Recent methods combining reduced representation library construction and next-gen sequencing (i.e., RADseq [6]) target an intermediate number of regions (shown schematically above). We expand on this approach to provide marker sets ranging from 100s to 100,000s of regions at low cost with no requirement of prior genomic data (ddRADseq; double digest RAD sequencing).

The genome serves simultaneously as a basic blueprint, encoding information for proper cellular and developmental processes necessary to produce an organism, and as a historical record of the demographic processes and selective forces acting in a given lineage. Exploration of mechanistic details through biochemistry, genetics, and development has lead to a deeper understanding of how genotype leads to phenotype, while exploitation of the historical record has enabled the fields of systematics, population genetics, and molecular ecology to elucidate the pressures and processes that shape diversity in populations and divergence between species. Studies of genetic information both encoded and recorded in genomes work with the same currency–comparison of homologous sequences across individuals–but these approaches employ very different modes of inference, and as such the details of a particular experiment dictate optimal marker resolution (Figure 1). To address the need for flexibility in marker number, we describe a next-generation sequencing-based method for determining individual sequence genotypes that can be tuned to sample a large range (from hundreds to hundreds of thousands) of randomly distributed regions genome-wide.

**Figure 2:**
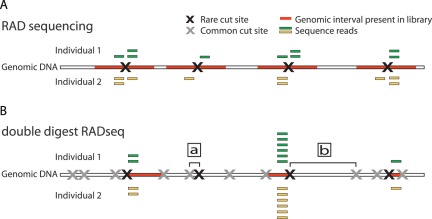
Double digest RAD sequencing improves efficiency and robustness while minimizing cost. (**A**) Traditional Restriction-Site Associated DNA sequencing (RADseq) uses a single restriction enzyme (RE) digest coupled with secondary random fragmentation and broad size selection to generate reduced representation libraries consisting of all genomic regions adjacent to the RE cut site (red segments). (**B**) Double digest RAD sequencing (ddRADseq), by contrast, uses a two enzyme double digest followed by precise size selection that excludes regions flanked by either [a] very close or [b] very distant RE recognition sites, recovering a library consisting of only fragments close to the target size (red segments). Representation in this library is expected to be inversely proportional to deviation from the size-selection target, thus read counts across regions are expected to be correlated between individuals (yellow and green bars).

The plummeting cost and skyrocketing throughput of DNA sequencing has begun to enable sequencing of entire genomes of study populations of some focal species [1], [2]; however, even in traditional model species (e.g., humans, laboratory mice, and *Drosophila*) resources for complete genome resequencing of large numbers of individuals by single investigators are still limited. As nearly all population and comparative analyses depend on an increasing number of individuals or samples for statistical power, several methods have emerged to increase the number of individuals sampled for the same resource investment by reducing the fraction of each individual genome sequenced. The crucial hurdle that must be overcome in reducing sampling for each individual is ensuring that the same (homologous) regions are examined between individuals. An early solution to this problem took advantage of sequence specificity of restriction endonucleases to construct a “reduced representation” sequencing library for polymorphism discovery [3]. While initially limited to SNP discovery rather than individual genotype determination by the cost and throughput of Sanger sequencing, later studies using a similar approach capitalized on high-throughput massively parallel sequencing such as 454 (454 Life Sciences, Branford, CT) and Genome Analyzer (Illumina, Inc., San Diego, CA) and reported both reliable SNP discovery and genotyping [4], [5]. Second-generation sequencing of DNA libraries comprised only of regions adjacent to restriction sites was later dubbed Restriction Associated DNA sequencing (RADseq; Figure 2A; [6] and developed further in [7], [8]). More refined methods have since emerged (e.g., Multiplexed Shotgun Genotyping [MSG]; [9]), but rely on having a complete reference sequence available. Subsequently, studies extended RADseq to species that lack a reference genome sequence, but have restricted variant discovery to only those regions that contained at most one or two polymorphic sites [10]–[12].

While these approaches permit genotyping of multiple individuals with substantially reduced sequencing investment, they are limited in their ability to allow researchers to tune the fraction of genome sampled (i.e., to genotype only the number of markers needed for a given experiment). Furthermore, while the RADseq method is suitable for systems that lack a sequenced reference genome, the existing computational tools for analyzing resulting data perform with relatively poor efficiency. In published examples of RADseq data analyzed without a reference genome [10]–[12], approximately half of the sequence data in each case was discarded because the analysis was not robust to error in sequence reads, and an additional ∼30–50% of loci were discarded due to the presence of more than 1–3 variable sites in each region. In addition to inefficiency, including only reads below a set number of nucleotide differences between haplotypes at a locus introduces bias in these data, removing rapidly diverging regions and complicating analyses such as phylogenetic rate and coalescence time estimation [13]–[16]. Thus, the ability to optimize the number of loci sequenced and maximize the number of sequence reads incorporated in the analysis, and to take advantage of multiple sites per locus would improve both the efficiency and utility of this approach.

To increase the breadth of RADseq applications, we have elaborated on the method described by Baird et al. [6] by eliminating random shearing and explicitly using size selection to recover a tunable number of regions, which are distributed randomly throughout the genome. Moreover, to maximize our ability to multiplex (i.e., increase the number of samples per sequencing lane), we also have developed a two-index combinatorial tagging approach (e.g., n * m individuals using *n+m* indices) and an accompanying computational analysis toolkit and lightweight data management component to facilitate high-order multiplexing of many hundreds of individuals. We also developed a graph clustering-based pipeline to maximize sequence read inclusion in analysis and permit detection of orthologous haplotypes regardless of divergence (i.e., without arbitrary similarity requirements), thereby improving analysis sensitivity and efficiency. Our software pipeline utilizes a novel approach for filtering resulting loci independent of coverage depth and converts the resulting haplotype multiple alignments into standard SAM/BAM format for downstream analysis, such as variant detection using the Genome Analysis Toolkit [17] or samtools [18]. This method has proven inexpensive (i.e., fractions of a penny per individual per site), rapid (i.e., approximately 8 hours of hands-on time), requires little starting material (i.e., 100 ng of DNA), and is suitable for high-throughput applications (all steps can be carried out in microtiter plates). In addition, this method can be employed, and its results efficiently analyzed, with no prior knowledge of genome sequence.

## Methods

### Double Digest Restriction Associated DNA (ddRAD) Sequencing

We have developed a protocol that builds on the RADseq method [19] but which differs in two principal respects (Figure 2). First, our method eliminates random shearing and end repair of genomic DNA (an advantage shared with a family of partially overlapping protocols such as MSG, CrOPS, and other recent RADseq derivatives [9], [20], [21]). Instead, we use a double restriction enzyme (RE) digest (i.e., a restriction digest with two enzymes simultaneously) that results in at least five-fold reduction in library production cost–complete ddRADseq libraries cost ∼$5 per sample, while the necessary enzymatic steps following the initial restriction digest and ligation in random shearing RAD libraries alone introduce a cost of ∼$25 per library (NEB, Ipswich, MA). Furthermore, the elimination of several high-DNA-loss steps permits construction of ddRAD libraries from 100 ng or less of starting DNA. Second, we introduced a precise selection for genomic fragments by size, which allows greater fine-scale control of the fraction of regions represented in the final library (see results). By combining precise and repeatable size selection with sequence-specific fragmentation, double digest Restriction-Site Associated DNA sequencing (ddRADseq) produces sequencing libraries consisting of only the subset of genomic restriction digest fragments generated by cuts with both REs (i.e., have one end from each cut) and which fall within the size-selection window (Figure 2B). This combination of requirements can be tuned to generate libraries consisting of fragments derived from hundreds to hundreds of thousands of regions genome-wide.

Precise, repeatable size selection offers two further advantages. First, because only a small fraction of restriction fragments will fall in the target size-selection regime (<5% in conditions described here), the probability of sampling both directions from the same restriction site is low. This reduces “duplicate” (i.e., immediately neighboring) region sampling, which effectively halves the number of reads that are required to reach high-confidence sampling of a SNP associated with a given RE cut site. Second, shared bias in region representation favoring fragments closest to the mean of size selection, in turn, biases independent samples (e.g., from different individuals) towards recovering the same genomic regions (Figure 2B). Because of this correlated recovery, regions are “filled in” with reads in approximately the same order across all individual samples, and samples with read recovery counts below saturation will still share a significant number of well-covered regions (“Experimental ddRADseq results” below; Analysis S1 Supporting Figure 4; Analysis S1 “Region recovery: ddRADseq vs. random shearing”). Both of these properties make the ddRADseq method robust to under-sampling with respect to read counts, which is a commonly observed problem arising from unequal read representation across individual samples in pooled sequencing experiments [9], [22], [23].

### Sample Multiplexing via Combinatorial Indexing

The double RE digest and precise size-selection of genomic fragments approach described here permits tuning of the number of regions recovered across several orders of magnitude (Table 1; “Results” below). As the per-base sequencing depth required for genotype determination is constant, the necessary sequencing investment for one individual or sample is inversely proportional to the total number of regions sampled. For example, if a combination of restriction digest and size selection efficiently recovered fragments derived from 10,000 regions genome-wide, an average of 20x coverage could be achieved at an investment of 200,000 sequence reads, which would permit sequencing of over 1000 individuals in a single Illumina HiSeq 2000 lane (based on common observation of average read counts exceeding 200 M reads per lane). Furthermore, as costs of library construction by double digest are five to ten fold less than random-shearing methods (due to the cost of shearing and enzymatic end-repair), construction and sequencing of individually indexed libraries for thousands of samples is financially feasible (i.e., a few dollars per individual). Thus, ddRADseq permits construction of highly multiplexed libraries, due to the ability to decrease read count requirements in sequencing and reduce cost per individual in library construction.

**Table 1:** Simulated region recovery and optimal per-individual read investment demonstrate marker set flexibility.

	Genome (GB)	SbfI-EcoRI	SphI-EcoRI	EcoRI-MspI	SphI-MluCI	NlaIII-MluCI
Human (*Homo sapiens)*	3.10	1 (0.01% 34)	10 (0.08% 249)	20 (0.19% 581)	40 (0.30% 915)	200 (1.49% 4619)
Rat (*Rattus norvegicus*)	2.72	1 (0.01% 24)	10 (0.08% 224)	20 (0.18% 492)	40 (0.35% 949)	200 (1.79% 4874)
Mouse (*Mus musculus*)	2.72	1 (0.01% 26)	10 (0.09% 248)	20 (0.20% 540)	50 (0.40% 1094)	200 (1.81% 4905)
Corn (*Zea mays*)	2.07	0.7 (0.01% 15)	6 (0.06% 128)	30 (0.33% 676)	30 (0.38% 777)	200 (1.99% 4114)
Lizard (*Anolis carolinensis*)	1.80	0.7 (0.01% 15)	7 (0.08% 150)	20 (0.31% 550)	20 (0.27% 488)	100 (1.61% 2904)
Zebrafish (*Danio rerio*)	1.41	0.4 (0.01% 8)	4 (0.06% 82)	10 (0.16% 230)	20 (0.31% 443)	100 (1.60% 2254)
Finch (*Taeniopygia guttata*)	1.22	1 (0.03% 31)	5 (0.08% 101)	8 (0.14% 174)	10 (0.26% 321)	100 (1.69% 2066)
Chicken (*Gallus gallus*)	1.11	0.9 (0.02% 18)	5 (0.10% 111)	7 (0.13% 148)	20 (0.41% 450)	100 (1.82% 2017)
Stickleback (*Gasterosteus aculeatus*)	0.46	0.3 (0.02% 7)	1 (0.06% 28)	7 (0.31% 143)	9 (0.40% 183)	50 (2.24% 1032)
Fugu (*Takifugu rubripes*)	0.39	0.3 (0.02% 6)	1 (0.06% 22)	5 (0.29% 114)	6 (0.32% 125)	30 (2.03% 796)
Limpet (*Lottia gigantea*)	0.36	0 (0.00% 0)	0.6 (0.04% 13)	3 (0.21% 78)	0.9 (0.05% 19)	10 (0.62% 225)
Fire Ant (*Solenopsis invicta*)	0.35	0 (0.00% 0)	1 (0.06% 22)	6 (0.35% 125)	2 (0.14% 48)	10 (0.83% 295)
Mosquito (*Anopheles gambiae*)	0.27	0 (0.00% 0)	0.9 (0.07% 18)	4 (0.36% 98)	4 (0.34% 91)	20 (1.71% 467)
Leech (*Helobdella robusta*)	0.24	0 (0.00% 0)	0.6 (0.05% 12)	1 (0.16% 39)	1 (0.15% 36)	10 (0.94% 223)
Honeybee (*Apis mellifera*)	0.23	0 (0.00% 0)	0.6 (0.06% 13)	5 (0.48% 109)	0.8 (0.08% 17)	6 (0.57% 131)
Fruitfly (*Drosophila melanogaster*)	0.17	0.05 (0.01% 1)	0.7 (0.08% 14)	3 (0.44% 73)	2 (0.29% 48)	10 (1.49% 250)
Thale cress (*Arabidopsis thaliana*)	0.12	0 (0.00% 0)	0.3 (0.06% 7)	2 (0.37% 44)	0.8 (0.15% 17)	8 (1.50% 179)

A single set of reagents coupled with appropriate digest (and sizing) conditions can be used to tune the number of recovered fragments over two orders of magnitude in most species (see text). Parameters for ddRADseq recovery simulations: “wide” automated size selection (300 bp±36 bp simulated with mean = 300 bp, SD = 18 bp; see Figure 4) with different RE combinations. The values in each cell report: the approximate number of fragments expected (in thousands), the fraction of the diploid genome that would be sampled in a 100 bp, paired end read (200 bp total), and the expected number of reads (in thousands) required to saturate the regions at >7× coverage.

Assigning each sequence read from a given experiment to one of hundreds (or even thousands) of individual samples is a substantial challenge. Previous studies have reported pooling strategies that resolve up to several dozen individuals in a sequencing lane using a “molecular barcode,” consisting of a short stretch of known sequence immediately adjacent to the genomic sequence read (12 barcodes were reported in [22]; 96 in [9]). When more than 12 individuals or samples are pooled, however, the cost of synthesizing the oligonucleotides used to make barcoded adapters for library construction presently exceeds the cost of a lane of Illumina sequencing. Thus, while in principle it is possible to expand the repertoire and sequence length of in-line barcodes to fit the required number of individuals, the requirement for one unique barcoded adapter (and therefore two DNA oligonucleotides) for each individual introduces substantial cost and logistical complexity.

Therefore, we developed adapters for ddRAD sequencing that simultaneously incorporate a combinatorial in-line barcode (per [22]) and a standard Illumina multiplexing read index (Protocol S1 Figure 1). In brief, a small number of barcoded adapters are ligated separately to individual samples in microplate format. These samples are then pooled following ligation, but before size selection. Size selection is performed on each pool of individuals and the resulting libraries are amplified with a primer that introduces an index that will be read off in a separate multiplexing read per the standard Illumina multiplexed paired-end sequencing protocol. Following PCR with uniquely indexed primers, multiple pools can be combined and individuals that share the same in-line barcodes (present in the adapter and detected as the first bases of the sequencing read) are distinguished based on the combination of adapter barcode and multiplexing read indices. This two-tier indexing scheme thus allows for an exponential increase in uniquely identifiable samples per pool, while avoiding additional oligonucleotide synthesis and sequencing costs associated with greater numbers of longer unique barcodes.

Here, we present oligonucleotide sequences for 48 uniquely barcoded ddRADseq library construction adapters as well as corresponding PCR primers for the 12 multiplexing read indices officially supported by Illumina analysis software (Sequences S1), but custom analysis permits the use of additional multiplexing read indices. The 48 adapter barcodes used in this work all differ by at minimum 2 base positions, which is sufficient to achieve >95–99% assignment of individual reads (Analysis S1 Supporting Table 1). All barcoded adapters and indexed PCR primer sequences provided generate standard Illumina sequencing libraries with respect to cluster generation and sequencing primers. Thus, no modifications to standard sequencing protocols are necessary after the completion of library construction. Furthermore, as all sequencing primer and flowcell annealing sequences are identical to those in standard Illumina multiplexing libraries, ddRADseq libraries can be sequenced in any combination of single-read or paired-end and with or without an Illumina-style multiplexing read (i.e., using only in-line adapter barcodes to distinguish samples, following [22]). This indexing approach is flexible and cost-effective, but the ability to pool large numbers of samples brings with it a need for tools to facilitate the analysis of these pooled data.

### Identification of Multiplexed Samples

To efficiently “de-multiplex” (match each sequence read to a single sample) this two-tier indexing scheme and to manage thousands of samples in a flexible, portable and lightweight Laboratory Information Management System (LIMS), we have developed a simple combination of Google Documents Spreadsheets and database tools in the Python programming language. We use this system both to track sample data and metadata (e.g., sex, population and pedigree information, phenotype) and to store and query multiplexing barcode and index data. We associate a single sample with up to four pieces of information: a flowcell designator, a lane number, the microplate well address of the adapter (and therefore adapter barcode), and optionally the multiplexing PCR primer index. This allows straightforward “de-multiplexing” of resulting data in the first phase of data analysis. Briefly, Illumina fastq format files (which have been processed into separate files based on Illumina multiplex read indices, if applicable) are further de-multiplexed by matching the first *k* bases of the read (where *k* is the length of the in-line barcode set, obtained from Google Spreadsheets LIMS) to the set of barcoded sequences expected in that lane (or in that multiplex read index set, from that lane). As all in-line adapter barcodes reported here differ by at least two positions, we retain any read that aligns, with one or fewer mismatches, to one and only one valid index and assign that read to the corresponding individual (and if desired, write that read data with quality to a new fastq file containing only reads from that individual). Together, this combination of inexpensive high-order multiplexing and free, familiar tools for tracking and resolving samples in a pooled sequencing experiment serves to substantially reduce barriers of cost and complexity for genome-scale analyses of large numbers of individuals.

### Polymorphism Discovery and Genotyping without a Reference Genome

Due to short read lengths and high error rates, methods for analyzing second-generation sequencing data generally require mapping sequencing reads to a fully sequenced genome from the same or a very closely related species (divergence must be low enough to expect that seed conditions for read mapping will be met even in rapidly evolving sequence, generally <1–5% divergence overall [24], [25]). In contrast to random shotgun libraries, in which reads are expected to start at all possible genomic positions, RADseq data consist of reads beginning only at restriction sites. As such, in the absence of error or polymorphism, the 10–100e^6^ reads generated by a single lane of parallel sequencing should represent no more than 1–100e^3^ unique sequences depending on enzyme choice, genome size and size-selection strategy. Thus, we have developed a *de novo* analysis strategy, which leverages this inherent reduction in data complexity to perform reference-free variant discovery and genotyping from ddRAD data.

### Short-Read Data Analysis

We begin by collapsing all identical sequences within a lane into a single record retaining the number of times the sequence was observed in each individual (based on barcoding and indexing), the average per-base quality across the sequence in all observations, and, if paired-end sequencing was performed, the associated unique paired-read sequences and counts. The resulting set of unique sequences consist of: single copy sequences with no nucleotide variation across sampled individuals, sequences representing segregating haplotypes of single-copy loci, high-copy or paralogous sequences, and error-containing reads. Previous RADseq unreferenced analyses have employed a variety of heuristic approaches to distinguish among these categories, such as discarding singleton reads to eliminate error-containing reads, grouping sequences that differ by 1–3 mismatches to identify sets of homologous alleles, and discarding homolog sets consisting of unusually large numbers of reads to eliminate paralog and interspersed repeats [10], [12]. These approaches are both inefficient (it is likely that an error that generates a singleton will occur at a non-polymorphic site and as such, the majority of error-containing reads are still informative) and arbitrarily restrictive, as insertions/deletions (indels), polymorphisms and multiple SNP haplotypes require extension beyond single-mismatch homology.

In place of individual heuristics for read trimming, ortholog inference, and paralog/repeat pruning, we employ a graph-based distance clustering approach to recover groups of maximally similar sequences followed by a novel “ploidy-aware” quality filter. We first compute pairwise distances between all unique sequences using BLAT [26]; while slower than short-read mapping approaches, this permits detection of more divergent haplotypes, including indel-containing regions. We then employ the MCL (Markov Cluster Learning) graph clustering algorithm to discover groups of unusually similar sequences, analogous to the OrthoMCL phylogenetic ortholog finding approach [27]. Next, we separately consider counts of all unique sequences from every individual in each cluster, and ask what fraction of reads report haplotypes beyond the ploidy of the organism under study (i.e., total counts of each unique sequence after the two most highly recovered in a diploid, one in a haploid, or four in a tetraploid). This results in a conservative estimate of either the fraction of error-containing sequences in a legitimate single-copy cluster, or the proportional representation of the top paralog in a cluster which groups sequences from more than one genomic region. Per-base error rates on the Illumina platform are generally 0.1–1.0%, therefore we expect 31*0.001–31*0.01 = 3.1–31% of 31 bp reads to contain an error. In the experiments reported here (see below), our per-base error rate ranged from 0.18–0.22%, suggesting approximately one in ten 31 bp reads are expected to contain an error, and we therefore discard any graph cluster consisting of more than 10% “non-first-two” sequences for each (diploid) individual; in other words, we retain only putative ortholog sets for which greater than 90% of reads were one of the two most frequent unique sequences in that set for each individual.

After assigning reads and filtering ortholog groups, we perform multiple alignments of all sequences in a group using MUSCLE [28]. Multiple alignment has the advantage of both correcting for register errors introduced early in individual reads, and maximizing the probability of correctly positioning indels between haplotypes [29]. Alignments are then written as reference-ordered SAM/BAM files [18] including @RG and @SQ headers, treating the most highly represented of the set of longest reads in each cluster as a pseudo-reference which is written to an accompanying reference fasta file. Converting ddRADseq clusters to SAM/BAM with preserved individual and sample metadata facilitates population-aware variant detection and genotyping on virtually all modern short-read analysis platforms, including samtools mpileup [18] and the Genome Analysis Toolkit (GATK) UnifiedGenotyper [17]. All new software described in this work is available at http://github.com/brantp/rtd.

## Results

### Implementing ddRADseq in an Emerging Model Rodent

We applied the double digest RADseq (ddRADseq) method for genotyping in an emerging model system, the deer mouse (genus *Peromyscus*). First, we developed and validated our method by genotyping ∼1000 segregating fixed differences in a cross between two sister species (*P. maniculatus* and *P. polionotus*). Second, we sought to genotype approximately 10,000 SNPs in natural populations of *P. leucopus* to test the utility of this approach in wild-caught samples. These proof-of-concept studies are described in detail below.

### RE Choice and Size Selection in Determining the Number of Sites to be Genotyped

To construct a high-density genetic map in a cross between sister species *P. polionotus* and *P. maniculatus,* we required genotype information for each animal in our cross at approximately 1,000 markers genome wide. We assumed a lower bound on the rate of fixed differences between G_0_ parents of approximately 0.001 [30], [31] and for sequence read lengths (and thus sampled region sizes) of 30 bp, we expected to sample a variable site (fixed between species) at a rate of one region in 30, which suggested a target set of 3e^4^ regions total. We estimated the appropriate set of REs and size-selection conditions to recover the appropriate number of genomic regions by performing simulations using the sequenced genomes of three distantly related rodents (laboratory mice [*Mus musculus*], rats [*Rattus norvegicus*], and the thirteen-lined ground squirrel [*Spermophilus tridecemlineatus*]; Analysis S1 “preliminary expectation”), which diverged from *Peromyscus* over 25 Mya [32]. As the results described below are consistent across all three comparisons, we report values only for *Mus*.

**Figure 3:**
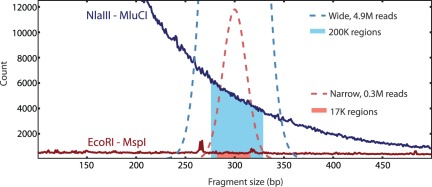
Double digest RAD sequencing provides flexibility in the number of homologous fragments recovered. Changing the restriction enzyme (RE) or size-selection regime modifies the fraction of genome recovered. **Simulation 1** (blue lines, shading): the expected fragment size distribution for a RE digest with NlaIII and MluCI (CATG and AATT) in the *Mus musculus* genome is shown (solid blue line). “Broad” size selection (300 bp±50 bp) is modeled by a normal sampling distribution (mean = 300 bp, SD = 25 bp). Under this sampling distribution, 4,900,000 sequence reads (dashed blue line) are expected to cover ∼119,000 regions at 7× or greater (blue area). **Simulation 2** (red lines, shading): the expected fragment size distribution for a digest with EcoRI and MspI (GAATTC and CCGG) is shown (solid red line). “Narrow” size selection (300 bp±24 bp; see text) is modeled by a normal sampling distribution (mean = 300 bp, SD = 11 bp; see Analysis S1 Supporting Figure 1). Under this sampling distribution, an investment of 315,000 sequence reads (dashed red line) is sufficient to recover ∼17,000 regions at 7× or greater (red area).

We surveyed several combinations of REs, seeking a pair that would yield between 1e^4^ and 5e^4^ fragments (targeting the 3e^4^ calculation above) when subjected to size selection appropriate for Illumina library preparation: a mean fragment size between 200 bp and 400 bp and the size-selection window not more than 50–100 bp wide [33]. We estimated 1.5e^6^ regions flanking cut sites for the enzyme EcoRI (both directions from each GAATTC sequence) in the *Mus musculus* genome (Ensembl release 61, NCBI M37), yet sampling only those sites that lie between 275 and 325 bases from a second cut site–MspI (CCGG)–is expected to yield a set of just 2e^4^ fragments (Figure 3; Analysis S1 “preliminary expectation”). Substitution of one or both REs for one with different recognition sequence frequencies was expected to modify the number of fragments recovered at a given size-selection window over a range of three orders of magnitude (see Table 1). Changing the size-selection window breadth within the range of constraints on sequencing libraries was expected modulate the number of fragments recovered over a range of approximately twofold (e.g., by doubling the size-selection window to 250–350 bp, 4e^4^ regions were expected to be included; Figure 3). Thus, simulations suggested that tuning two parameters–choice of REs and size-selection window–should permit optimal marker number recovery.

As current “second generation” sequencing technology is subject to relatively high error rates (in excess of 0.1%), it is necessary to sequence each base several times to produce confident genotypes (in our experience and concordant with [34], greater than 7× coverage is generally required). To achieve at least this level of coverage in 1.5e^4^–2e^4^ regions size selected from 275 to 325 bp in an EcoRI-MspI digest of the *Mus* genome (see above; Figure 3), we aimed for an average of 10x coverage, which corresponds to between 2e^5^ and 5e^5^ reads for each individual. Based on this estimate and an expected yield of 2.5e^7^ reads per lane of Illumina GAII sequencing, we prepared pools of 48 separately barcoded individuals per sequence lane.

### Experimental ddRADseq Results

To evaluate the performance of this approach, we prepared libraries as described (Analysis S1 “library construction”) and tested three methods of size selection. First, we attempted standard agarose gel electrophoresis on 2% agarose gels followed by excision of a band corresponding to the 300 bp; we prepared 48 individuals in six agarose gel lanes. Second, we tested the impact of changes in size-selection range and the efficacy of automated *versus* manual DNA size selection using automated size-selection technology, Pippin Prep (Sage Science, Beverly, MA; 2% agarose cartridge). We produced one 48-individual library in four lanes using automated size selection set to “narrow” setting with a mean of 300 bp and range of ±24 bp (276 bp–324 bp; the narrowest achievable range for this size mean) and a similar library under a “wide” setting with a mean of 300 bp and range of ±36 bp (264 bp–336 bp). Bioanalyzer (Agilent, Santa Clara, CA) results suggested that automated size-selection libraries were substantially more consistent than gel extraction libraries, but that we were generally able to achieve peaks within 10 bp of the expected size using gel excision (data not shown).

Three Illumina GAII sequencing lanes averaged 21.7 M reads, and after resolving reads by individual barcode (described above), individuals averaged 440 K reads (see Table S1). As divergence between *Peromyscus* and a closely related species with a fully sequenced genome (the house mouse *Mus musculus*) is substantially greater than the approximately 5% maximum nucleotide divergence for mapping short reads to a reference sequence (only 28.1% of 84 bp *Peromyscus* sequences are assigned to unique positions in the *Mus musculus* genome by BLAT [26], sequences with unique matches average 61% identity), the data were analyzed as described in “Methods; polymorphism discovery and genotyping without a reference genome”. We observed that individuals receiving >200 K reads in the narrow size-selection condition saturated at an average of 14–17 K shared regions (Analysis S1 Supporting Figure 2A, B). In the wide size selection, this saturation required 400 K reads and reached an average of 20–24 K shared regions (Analysis S1 Supporting Figure 2B, C). For automated size-selection libraries, region coverage was highly correlated between samples (r^2^∶0.71–0.93). The results demonstrate that it is straightforward to design an experiment (i.e., choose REs) targeting a given number of regions using an approximate expectation for cut site frequency and nucleotide variability, and then to precisely tune resulting recovery by modifying size selection. In contrast to automated size-selected samples, gel excision samples did not appear to saturate in the range of coverage observed. This is likely because size selection was imprecise or “leaky”, with substantial representation of fragments of lengths relatively distant from the size-selection target mean. While this prevents fine-tuning in the size-selection step, gel excision samples nevertheless exceeded 14 K regions for upper-quartile read sampling (>400 K reads per individual) indicating that careful practitioners can achieve roughly 50% of the precision and repeatability of automated DNA size selection.

### Comparison of Observed and Simulated ddRADseq

**Figure 4:**
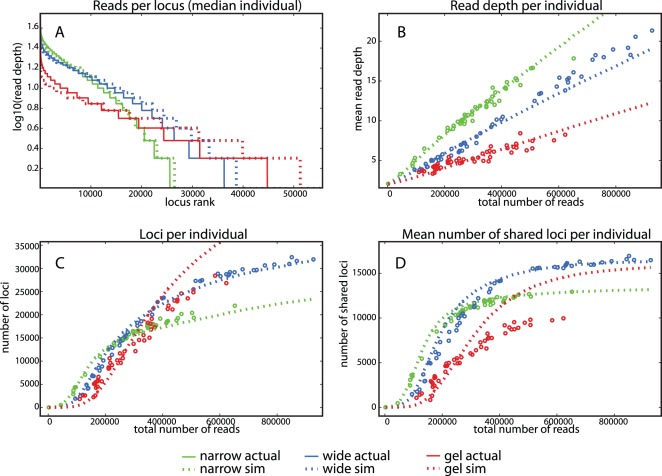
Recovery of genomic regions in deer mice (*Peromyscus maniculatus* and *P. polionotus*) is well predicted by simulation based on the laboratory mouse (*Mus musculus*) genome with precise size selection. Simulated data based on the *Mus musculus* genome (dashed lines) and actual data from a distantly related rodents *Peromyscus maniculatus* and *P. polionotus* (solid lines), both fragmented with EcoRI and MspI recognition sites. Sampling from the *Mus* genome is drawn from a normal distribution (mean = 300 bp and SD = 11.5, 17.5, and 30), which represents the best match for *Peromyscus* ddRADseq with size-selection windows of ±24 bp (“narrow”, green), ±36 bp (“wide”, blue) and ±25–50 bp (“gel”, red) respectively. The narrow and wide selection sets are based on a more precise automated size-selection method (PippinPrep, Sage Science). Recovery in ddRADseq experiments, both within and across individuals, is highly predictable: (A) Region coverage is highly correlated between simulated *Mus* and observed *Peromyscus* data. Simulations show good fit to automated size selection (median samples from each sizing strategy and simulation of matched read counts, r^2^ 0.99 and 0.98 for narrow and wide sizing, respectively), but match less well for gel extraction (median r^2^ 0.94). (B) Simulated data are concordant in mean sequence coverage across fragments as a function of total read depth per individual in all size-selection schemes (open circles: observed data, dotted line: simulation). (C) The number of regions with coverage ≥7× as a function of total read depth per individual, and (D) mean number of regions with coverage ≥7× shared with other individuals, show very high concordance with normal sampling distributions in both narrow and wide automated size selection but are less well fit by any tested sampling distribution for the gel extraction method.

We evaluated the accuracy of original estimates using simple simulation modeling of these experiments with our three size-selection regimes. We approximated size selection as a simple model of normally distributed sampling with mean 300 bp and unknown variance from the observed fragment size distribution derived from an *in silico* RE digest of the *Mus* genome with EcoRI and MspI (see above). We tested goodness-of-fit (Pearson r^2^ of log-transformed coverage across regions) of our *Peromyscus*-derived data for fragment coverage against that calculated from simulations with size-selection sampling distributions (SD = 1 bp 100 bp). Using best-fit size-selection sampling distribution parameters (mean = 300 bp, SD = 11.5 bp, 17.5 bp and 30 bp for narrow automated size selection, wide automated size selection and manual gel excision, respectively), we evaluated simulated region recovery and our real data with respect to: mean coverage, number of total regions covered at or above 7×, and the average number of regions shared between that data point (real individual or simulation result) and all others where presence in both is defined as coverage at or above 7× (Figure 4). For both automated size-selection conditions, all measured properties in real *Peromyscus* data were extremely well captured by *Mus* simulation results, indicating that with precise size selection, recovery in ddRADseq experiments both within and across individuals is highly predictable from genomes with even >40% average sequence divergence (see above) Thus, information from the genome of a related species (with similar base composition) can produce accurate estimates of required sequencing effort, thereby minimizing “over sequencing.”

Simulation results predict the observed sharp saturation of new regions recovered after approximately 200,000 reads in the “narrow” automated selection and 400,000 reads in the “wide” sizing conditions. This effect is not a result of averaging shared regions across individuals that received fewer reads overall (Analysis S1 Supporting Figure 2; Analysis S1 “modeling simulation”). Instead, read and region counts at which this saturation is observed in real data correspond well with the transition from logistic to asymptotic accumulation of new regions with additional sequencing investment in simulations (Figure 4C; Analysis S1 Supporting Figure 3; Analysis S1 “modeling simulation”). This saturation represents the optimal investment of sequencing resources for a particular combination of target genome, RE digest and size selection, since sequencing beyond this point primarily recovers poorly sampled regions unlikely to be shared among individuals. By performing simulations using the “narrow” and “wide” sampling models trained from our *Peromyscus* EcoRI - MspI data, we similarly can predict the saturation point in region counts and corresponding required read depth for fragment distributions resulting from *in silico* digest of any combination of target genome and restriction enzymes.

### Validation of ddRADseq Derived Genotypes in a Laboratory Cross

**Figure 5:**
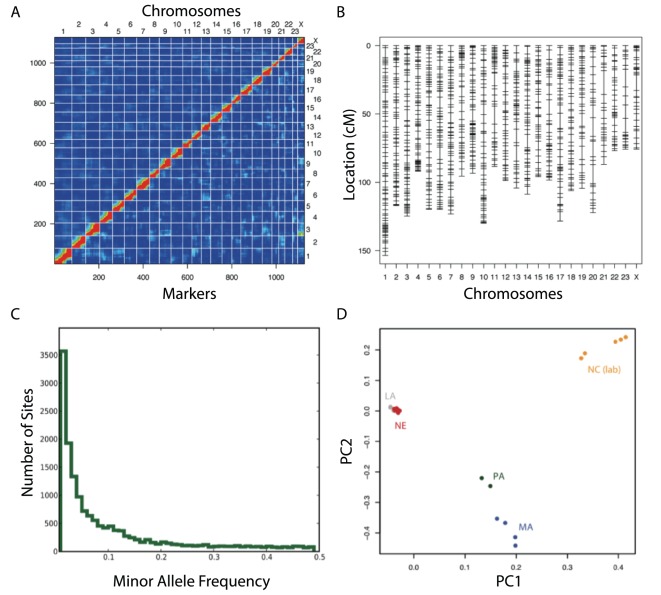
Discovery and genotyping of ddRADseq markers in a laboratory cross and wild populations without a reference genome. ddRADseq was used to identify SNPs between two *Peromyscus* species, neither of which had a genome sequence available, that were crossed as part of a QTL experiment. This yielded 1158 unique markers that were fixed within, but different between, the parental species. By calculating the fraction of recombinant genotypes and LOD of linkage between markers, we generated (**A**) 24 groups of strongly linked markers, heatmap colors represent strength of linkage in both recombination frequency (upper left) and LOD (lower right) between all pairs of markers; and (**B**) a genetic map with average inter-marker distance of 1.6 cM. ddRADseq was also used to genotype wild-caught and lab-reared individuals of *P. leucopus*. Our ddRADseq method permitted successful genotyping of wild-caught individuals even when the allelic variants within a population are unknown. (**C**) Estimated site frequency spectrum of a wild population of *P. leucopus* caught in a single Louisiana population. (**D**) Genetic structure between five populations of *P. leucopus*. Dots represent individuals (N = 92) and color indicates the states from which individuals were collected: LA = Louisiana; NE = Nebraska; PA = Pennsylvania; MA = Massachusetts; NC = North Carolina.

We produced the remaining ddRADseq libraries for all purebred parents, a single F1 individual and 192 F2-backcross progeny using the EcoRI/MspI enzyme pair and “wide” size selection scheme described above to complete our sampling of 2e^4^–3e^4^ regions from each animal with the goal of identifying and genotyping ∼1000 diagnostic SNP markers. We sequenced all libraries on an Illumina GAII and analyzed sequence reads as described (see “Methods”); all analyses reported here use genotypes from the GATK UnifiedGenotyper [17] with parameters QD (quality-by-depth) ≥6 and GQ (genotype quality) ≥20 based on optimization in other applications (data not shown). Because loci that are different between, but fixed within, each parental species are most informative for QTL analyses, we screened for markers that met these criteria, and that we could infer diploid genotypes for at least 150 (of 192) individuals in our cross. This filter produced 1,886 SNPs in 1,638 unique sequence regions. We estimated the genotype frequencies of each marker across all F2 progeny, and also the fraction of recombination events and LOD score between all marker pairs (Figure 5A). By varying the maximum fraction of recombination and minimum LOD score allowed among markers on a single linkage group, we constructed a linkage map using R/qtl [35] that contained 1,158 SNP markers in 24 linkage groups, consistent with the *P. maniculatus* karyotype, with a total length of 1,759.7 cM and an average inter-marker distance of 1.6 cM (Figure 5B). Our ability to construct a well-resolved genetic map of total map length similar to published high-density genetic maps for *Mus* [36] and associating the majority of genotyped sites with a number of linkage groups matching the *P. maniculatus* karyotype suggests that the ddRADseq approach efficiently generates high quality genotypes for laboratory crosses.

### *De novo* Analysis of ddRADseq Data in Outbred Populations

While library construction and sequencing for outbred wild population samples can be performed in a manner equivalent to that described for a laboratory cross, thorough analysis of resulting data in the absence of a reference genome is substantially more challenging due to the potentially much greater haplotype diversity at each locus in the recovered region set. Because our *de novo* sequence analysis pipeline is designed to be able to operate at greater sequence divergence between haplotypes at each genomic region, we tested our approach by performing genome-wide SNP genotyping in wild populations of *Peromyscus*. We first prepared two ddRADseq libraries containing a total of 54 wild-caught *Peromyscus leucopus* individuals collected from a single population in Louisiana. Libraries were prepared, sequenced, and analyzed according to the protocol described with the EcoRI/MspI enzyme pair, and “wide” size selection conditions as above (see “Methods”; Analysis S1 “Genotyping in a wild population”). We sequenced these libraries in two GAII lanes. Our *de novo* analysis pipeline recovered 6,199 variable regions, and yielded 15,962 total polymorphic sites with genotypes for at least 70% of individuals. We next calculated the distribution of minor allele frequencies (site frequency spectrum) for this population, which demonstrated consistent recovery of common variants and the expected roughly exponential distribution of rare alleles (Figure 5C).

We further explored the applicability of ddRADseq-derived markers in outbred samples by estimating structure among several *P. leucopus* populations. We generated ddRADseq libraries as above for a total of 92 individuals collected from four wild populations (Louisiana, Nebraska, Pennsylvania and Massachusetts, see Analysis S1) and a laboratory population (derived from North Carolina). Our analysis returned 18,907 SNPs from 7,435 orthologous fragments when we required 70% completeness. We used these SNPs to run a genetic principal component analysis (PCA) using the statistical software package, Eigensoft 3.0 [37] and found 7 significant eigenvectors (first two shown in Figure 5D). Genetic principal components support the expected geographic isolation as the dominant source of structure among these samples.

These applications of the ddRADseq approach not only demonstrate its success in species lacking a complete genome sequence, but also highlight a key advantage to our MCL clustering analysis: fewer than 20% of the informative SNP markers used in the outbred population analyses described above reside in single-SNP regions, and less than half in <3 SNP loci, which would have rendered the majority of that dataset unavailable to previous reference-free methods such as Stacks [38] as employed by analyses to date [10]–[12]. By not requiring a threshold identity for assignment of homology in our *de novo* analysis, we were able retain more sequence data, avoid bias against rapidly diverging or polymorphic regions, and incorporate longer reads than otherwise would have been possible.

## Discussion

Here we describe a combination of laboratory and computational methodology to permit highly repeatable and tunable recovery of hundreds to hundreds of thousands of randomly sampled regions from a target genome. In comparison to traditional RADseq methods, ddRADseq library preparation is less expensive and rapid (<8 hours hands-on time for dozens to hundreds of samples), completely compatible with microplate format, and can be performed using limited amounts of genomic material (<100 ng). Furthermore, due to the removal of random shearing (and therefore random recovery), correlated recovery of regions across individuals results in increased robustness to variability in read count (see “Results”; Analysis S1 Supporting Figure 4). As sequencing depth required to reach saturation is a direct function of the number of regions sampled (Table 1), the number of individuals which can be genotyped in a single sequencing lane is inversely proportional to the number of regions recovered. For example, we chose to recover 15–25 K regions in one experiment described here, for which saturation was achieved at less than 500 K reads per individual.

### Combinatorial Multiplex Indexing

For experiments other than genome-wide association studies, whole-genome scans for selection and population differentiation, recovery of tens of thousands of regions is often sufficient. Our simulations suggest that per-sample investment of less than 1 M reads in the appropriate digest and size-selection strategy is sufficient to achieve coverage enabling confident genotype determination at such region counts, which means that with modern sequencing capacity (20–200 M reads per lane, depending on technology) dozens to hundreds (and potentially thousands) of individuals can be pooled in a single sequencing lane. To facilitate inexpensive construction of libraries with large numbers of individuals, we developed a combinatorial indexing scheme that requires no modification to standard Illumina sequencing. Our applications of combinatorial indexing have combined ≥192 samples in a single Illumina HiSeq lane, with a recent example lane yielding 167 M reads in total amongst 192 individuals with median individual read count of 0.7 M reads and interquartile range of 0.3 M–1.2 M reads. Coefficients of variation across all pooled-sampled sequencing lanes range from 0.4–0.8 for all experiments performed to date. Thus, generating an average of double the desired minimum read count has proven sufficient to completely cover most or all samples. To simplify the process of handling sequence data generated from hundreds of pooled individuals we have implemented a lightweight LIMS for tracking and de-multiplexing samples based on the freely available Google Documents Spreadsheet platform.

### Reference-free RADseq Analysis by Graph Clustering

Analysis of RADseq data in the absence of a reference genome has been performed in a small but growing collection of studies employing the open-source Stacks package [38]. Stacks includes a full suite of tools for tracking pooled samples and performing the “off-by-N” assignment of alleles to a locus described above, as well as sample-by-sample genotyping and data storage. While the Stacks package is a complete and robust solution tailored to *de novo* analysis of random shearing RADseq data, we were motivated to develop our own implementation for four principle reasons. First, we wished to avoid an arbitrary sequence distance threshold between alleles for a single locus, as described above. Second, Stacks filters paralog and high-copy loci by read coverage, assuming random coverage across loci; this assumption is violated by the correlated recovery observed in ddRADseq, necessitating development of the ploidy-based filter (see “Methods”). Third, to increase efficiency of ddRADseq *de novo* analysis we sought to incorporate error-containing reads in our analysis rather than filtering these at the outset. Fourth, we wanted to be able to take advantage of recent improvements in statistical methods for genotype calling from short read data, such as multiple sample genotyping in the GATK program [17]. Our computational pipeline for genotyping in the absence of a reference genome achieves approximately 30–50% higher sensitivity in read incorporation than observed in the previously described applications of Stacks (62–75% of reads fall into well-recovered regions; greater than 68% for all automated size-selection samples, compared to approximately 50% [10]). Because error, frameshift, and low-quality containing reads are incorporated by the clustering process, our *de novo* analysis approaches the efficiency of read incorporation observed in reference-mapping approaches such as BWA (∼80% mapping for 32 bp reads [39]). In addition, graph clustering permits grouping of haplotypes with any number of mismatches, provided the global similarity relationships among all reads support significant sequence homology between them; for instance, in an outbred wild population this increased sensitivity by five fold over single-mismatch haplotype pairing. Our analysis also produces standard SAM/BAM formatted alignments that retain sequence read quality scores. This feature permits employment of quality-adjusted metrics in variant detection (such as quality-by-depth in the Genome Analysis Toolkit [17]) to prevent reduction of specificity in resulting genotype data, and facilitate accurate genotype determination even at relatively low read investment (>7×; [34]). We combined these features to simultaneously discover and genotype thousands of fixed differences in a laboratory cross and tens of thousands of SNPs in wild population samples for ∼$20 per sample total (<$5 sample prep, $15 sequencing) on the Illumina GAII platform and well under $10 (<$5 sample prep +<$5 sequencing) on the Illumina HiSeq 2000 platform.

### Conclusions

The ddRADseq method described here, in conjunction with huge strides in both the throughput of sequencing (e.g., Illumina HiSeq 2000) and in genotype analysis based on short read sequence data (e.g., GATK UnifiedGenotyper, samtools) permits high throughput simultaneous discovery and genotyping of sequence polymorphism either with or without an existing reference genome. Compared to existing RADseq approaches, ddRADseq permits greater flexibility and robustness in region recovery, and a substantial decrease in cost, required genomic material from samples and researcher time investment. Here, we provide a detailed protocol for the laboratory methods as well as an open-source computational pipeline (based on freely available software), which we hope will make this method accessible and widely applied to a range of biological problems in a diversity of organisms.

## Supporting Information

Protocol S1 **Detailed Protocol.** Complete laboratory protocol for design and execution of ddRADseq studies. The up-to-date protocol is also available at http://www.bit.ly/ddRAD.
(DOC)

Sequences S1 **Oligonucleotide sequences.** This Microsoft Excel spreadsheet documents all sequences of PCR primer and adapter oligonucleotides for experiments described in this work.
(XLS)

Analysis S1 Additional details on simulations described in this work. Also includes parameter values and run conditions for proof-of-concept analyses in laboratory cross and wild population experiments.
(PDF)
