# Supplementary material for: Double Digest RADseq: An Inexpensive Method for De Novo SNP Discovery and Genotyping in Model and Non-Model Species
Source: PLoS One. 2012 May 31;7(5):e37135. doi: 10.1371/journal.pone.0037135 (PMC3365034; doi:10.1371/journal.pone.0037135)
Supplement: Protocol S1 — Detailed Protocol. Complete laboratory protocol for design and execution of ddRADseq studies. The up-to-date protocol is also available at http://www.bit.ly/ddRAD. (DOC) [file pone.0037135.s001.doc]

Double Digest RADseq: an inexpensive method for de novo SNP discovery and genotyping in model and non-model species

Current version of this protocol resides at <http://www.bit.ly/ddRAD> and should be used when possible.

**ddRAD PROTOCOL**

BRIEF GLOSSARY

Adapter: fully or partially double-stranded product of annealing two oligos. Adapters are ligated to genomic DNA at restriction enzyme cut sites in order to add barcodes and common PCR priming sequences.

Barcode: short DNA sequence downstream of the sequencing primer annealing region of an adapter. Used to resolve products of different ligation reactions (usually separate individuals) after sequencing pooled libraries. Note that when selecting adapter barcodes to combine for Illumina sequencing of pooled samples, it is important to ensure approximately balanced base composition (equal ratios of A,C,G,T in each barcode position) to facilitate cluster detection.

Fragment: section of genomic DNA resulting from restriction enzyme cleavage.

Index: short DNA sequence introduced during PCR amplification of the final library that uniquely identifies products of that PCR reaction. Used combinatorially with Adapter P1 barcodes to resolve multiplexed sample pools.

Library: a collection of sequencing-competent fragments.

**Figure 1. Diagram of oligos and adapters; final library:**


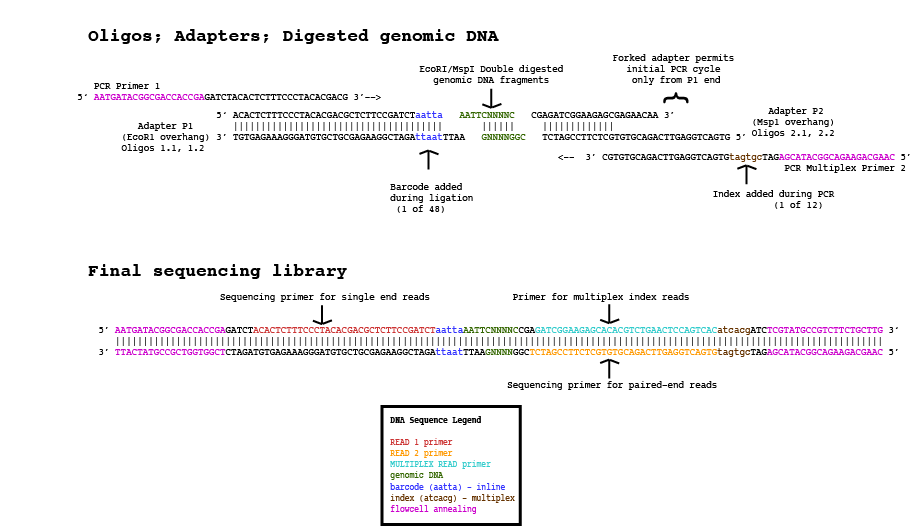


MATERIALS AND REAGENTS

*Digestion, Ligation & PCR*

- Restriction endonucleases (RE) and a buffer that is compatible with both REs

- Adapter P1: annealed oligos 1.1 and 1.2 (1 pair per barcode)

- Adapter P2: annealed oligos 2.1 and 2.2 (common to all barcodes and indices; biotinylation of oligo 2.2 optional)

- Annealing buffer stock (10X):

100 mM Tris HCl, pH 8

500 mM NaCl

10 mM EDTA

- T4 DNA ligase and buffer

- Pippin Prep reagents and cassettes

- PCR primer oligos: “PCR1” common to all reactions; “PCR2” primers contain unique indices that allow for multiplexing of multiple PCR amplification pools in a single Illumina lane

- Phusion PCR kit (Finnzyme or NEB)

*AMPure XP Bead & Dynabead Cleanups**

- AMPure XP beads (Beckman Coulter Genomics)

- Dynabeads® M-270 Streptavidin (Invitrogen)

- Dynabead binding and washing (B&W) Buffer (2X):

10 mM Tris-HCl (pH 7.5)

1 mM EDTA

2 M NaCl

- Rare earth magnets and/or 96-well Ampure magnetic plate

- Elution solution/buffer for AMPure beads (water, TE, Qiagen buffer EB, etc.)

- Freshly diluted 70% ethanol

**Biotinylation of adapter P2 is required for streptavidin bead cleanup. This step is optional (see “Streptavidin bead removal of ligation products with adapter P1 on both ends” below)*

**ASSOCIATED ONLINE DOCUMENTS**

[ddRAD Experimental Design](https://docs.google.com/document/d/1QY0LndEUywyGmOBCF7xDQeI_f5MNB_T3lKwSSvbFtX4/edit) - guide to selection of restriction enzymes and size selection conditions

[ligation molarity calculator](https://spreadsheets.google.com/spreadsheet/ccc?key=0AnHEwF1NpAxDdGtJdTRxVEZ1R3diY3hFV3p1Z3FkWWc&hl=en_US) - guide for calculating appropriate adapter concentration for ligations

[ddRAD oligo table](https://spreadsheets.google.com/spreadsheet/ccc?key=0AnHEwF1NpAxDdEFnWXBEMy02Y2V2UGw4OEJ3SWFtVGc&hl=en_US) - spreadsheet of adapter and PCR oligonucleotide sequences referenced in this protocol

**BENCH PROTOCOL**

**Anneal Adapters**

Single-stranded oligos need to be annealed with their appropriate partner before ligation. We provide sequences for 48 uniquely barcoded adapter P1 oligo pairs (oligos 1.1 and 1.2) and common adapter P2 (oligos 2.1 and 2.2), see [ddRAD oligo table](https://spreadsheets.google.com/spreadsheet/ccc?key=0AnHEwF1NpAxDdEFnWXBEMy02Y2V2UGw4OEJ3SWFtVGc&hl=en_US).

1. To create Adapter P1, combine each oligo 1.1 with its complementary oligo 1.2 in a 1:1 ratio in working strength annealing buffer (final buffer concentration 1x) for a total annealed adapter concentration of 40uM (for example, if purchased oligos are resuspended to an initial concentration of 200uM, use 20ul oligo 1.1, 20ul oligo 1.2, 10ul 10x annealing buffer and 50ul nuclease-free water). Do the same for oligos 2.1 and 2.2 to create the common adapter P2.

2. In a thermocyler, incubate at 97.5°C for 2.5 minutes, and then cool at a rate of not greater than 3°C per minute until the solution reaches a temperature of 21°C. Hold at 4°C.

3. Prepare final working strength concentrations of annealed adapters from this annealed stock (the appropriate working stock dilution for your experiment can be determined from our [ligation molarity calculator](https://spreadsheets.google.com/spreadsheet/ccc?key=0AnHEwF1NpAxDdGtJdTRxVEZ1R3diY3hFV3p1Z3FkWWc&hl=en_US)). For convenience, it is possible to store the adapters at 4°C while in active use.

**Double Digest**

Consult product information for your chosen restriction enzymes regarding digest conditions.

1. Double digest 100-1000 ng of high quality genomic DNA with selected restriction enzymes, in a 20-50ul reaction volume. Use a digestion buffer appropriate for both enzymes. (Note: This protocol is expected to work with as little as 100ng of genomic DNA, though we typically digest between 200-500 ng of DNA.)

2. Run the digestion as appropriate for the chosen REs. To ensure complete digestion, we run our double digests for 3 hours at 37°C, holding at 4°C. Do not heat kill the enzymes, as this may skew base composition in the resulting fragment library. Before proceeding with step 3, cool the reaction to room temperature. Alternatively, reactions can be stored at 4°C overnight.

3. Clean the double digest with AMPure XP beads following the provided protocol, using a magnetic plate (SPRIPlate Super Magnet Plate) or rare earth magnets and a tube rack (see photo) to separate beads from the solution. (Note: The Ampure XP protocol recommends using a volume of beads equal to 1.8X the volume of the solution being cleaned; we have had good success using 1.5X volume of beads, and recommend this ratio for all steps prior to PCR amplification).

4. Quantify the concentrations of your cleaned digests. Fluorometric (Spectramax plate reader or Qubit [Invitrogen]) measurement of DNA concentration is highly recommended; these measurements will be used to for preparing ligations.

**Adapter Ligation**

Ligation efficiency depends on the total number of adapters and the number of fragment ends in a digested sample. We find that a 2- to 10- fold excess of adapters to complementary sticky ends produces efficient ligations. To assist in calculation of dilutions from annealed stocks (“Anneal Adapters” above) and ligation conditions, it is useful to save a copy (File->Make a copy) of our [ligation molarity calculator](https://spreadsheets.google.com/spreadsheet/ccc?key=0AnHEwF1NpAxDdGtJdTRxVEZ1R3diY3hFV3p1Z3FkWWc&hl=en_US) in your own google docs account, and edit the fields shaded in green to suit your experiment depending on whether you are estimating adapter ligation concentrations from an *in silico* experiment (first worksheet) or from single/double enzyme cuts run on a Bioanalyzer (second worksheet). Note that annealed adapters in the molarity calculator are set to dummy values of 4uM (4 pmol/ul) concentration corresponding to the 10-fold dilution of the 40uM annealed adapter stock solution from the annealing reaction described above. If a different annealed adapter stock concentration is used, edit values in row 16 of this spreadsheet accordingly. The ligation conditions for a 40ul reaction are listed below; however, larger ligation reaction volumes can be used if needed and the reagents should be modified accordingly.

In a 40ul reaction volume:

1. Combine the appropriate amounts of working stock dilution of adapters P1, P2 and digested DNA. Standardizing the amount of digested DNA included in each ligation per sample concentrations measured above in “Double Digest” step 4 facilitates ligation reaction set-up and enables direct pooling prior to size selection.

2. Separately, create a master mix of T4 DNA ligase, T4 DNA ligase buffer, and water. (Note: the universal P2 adapter can be added to the master mix instead of individual reactions [step 1] for convenience). Each 40ul ligation should contain an amount of T4 ligase appropriate for the quantity of DNA to be ligated (see ligase unit definition in NEB documentation) and 4uL of 10X ligase buffer.

3. Combine the digested DNA and adapters with the appropriate amount of master mix. Add water to total reaction volume 40uL.

4. Incubate at room temperature (23°C) for 30 min, then heat-kill at 65°C for 10 min. After the heat-kill, cool the solution at 2°C per 90 seconds until it reaches room temperature.

**Pooling prior to size selection**

Samples individually barcoded with a unique P1 adapter can be pooled after the ligation step and cleaned with AMPure XP beads. The end goal is to pool every uniquely barcoded individual into a final 30ul sample such that the entire pool of ligation products can be loaded onto a Pippin Prep for size selection. The number of individuals that can be pooled into the final 30ul for size selection depends on the number of unique P1 adapters used. Combining large numbers of individuals may require multiple bead cleanups as follows:

1. Based on the total amount of pre-ligation genomic DNA in each sample, combine equal amounts of ligated DNA (now barcoded) from each sample to create a pool of individuals, total DNA mass ≤ 15ug (if samples were standardized per step 1 of “Adapter Ligation” above, equal volumes of each sample can be pooled). For large numbers of individual samples, the resulting volume of the pooled library will be large (e.g. assuming 48 uniquely barcoded P1 adapters were used, the final pooled amount would be 48 indviduals * 40ul ligation = ~2ml). This pooled volume should be split equally into 1.5ml microcentrifuge tubes such that each aliquot does not exceed 400ul (e.g. 6 aliquots from 1920ul [40ul*48individuals] = 320ul each).

2. Clean pools or pooled aliquots following the standard AMPure XP bead protocol using a 1.5x ratio of bead solution. This bead ratio will efficiently remove short DNA fragments such as unligated adapters and adapter-adapter ligation products. If the pooled library volume is small enough that it does not require multiple aliquots, elute the pool in a final volume of 30ul--this amount can be directly loaded onto the Pippin Prep cassette (see next section). If the pooled library volume was large enough to be split into multiple tubes as discussed in step 1, elute each AMPure cleanup in 40ul, combine elutions and perform a final AMPure cleanup, eluting in 30ul.

**Size Selection with Sage Science Pippin-Prep**

Although gel extractions can recover fragment sizes appropriate for Illumina sequencing, automated DNA size selection provides far superior results (e.g. Sage Science Pippin Prep [http://www.sagescience.com](http://www.sagescience.com/)).

Note that when specifying target range for size selection, it is crucial to account for the additional 76bp of adapter DNA added in ligation.

**Streptavidin bead removal of ligation products with adapter P1 on both ends (*optional*)**

Although only fragments ligated to both P1 and P2 Adapters will cluster during sequencing, fragments with P1 Adapters on both 5’ and 3’ ends will still amplify in the subsequent PCR step. If the final sequencing library contains many fragments with two adapter P1 ends, then it becomes difficult to quantify how much sample to load on the sequencer for optimal cluster generation. Fragments with only adapter P1 ends can be removed from libraries using Streptavidin-coated Dynabeads and biotin-labeled P2.2 oligos.

After the size-selection step, follow the standard Invitrogen protocol for both bead preparation and hybridization of beads with biotin-labeled DNA. We recommend following step 2.1.4 “Immobilization of Nucleic Acids”, with 3 washes and resuspension of beads in elution buffer.

**PCR Amplification to Generate Illumina Sequencing Libraries**

To add Illumina flowcell annealing sequences, multiplexing indices and sequencing primer annealing regions to all fragments (see fig 1) and to increase concentrations of sequencing libraries, we perform a PCR amplification with a Phusion™ Polymerase kit. For large-scale combinatorial multiplexing of samples, we include a set of 12 uniquely indexed PCR2 primer sequences. Each of these primers will add a unique index sequence to all fragments in the PCR. Therefore, it is possible to uniquely label 576 individuals [48 (adapter P1 barcodes) x 12 (PCR2 indices)], to combine in a single lane of sequencing.

For each Pippin Prep elution or gel extraction, set up 4-8 PCR reactions in 20ul total volume:

1. For each PCR, combine ~20ng of size-selected sample, PCR primers 1 and 2 at final concentration 2uM each, and the recommended amount of 5X-HF buffer, dNTPs, water and Phusion polymerase in a standard 200ul PCR tube.

2. Run 8-12 cycles of PCR at the recommended Phusion conditions. Increasing cycle number beyond this can introduce substantial (>1%) base mis-incorporation and exacerbate size and composition bias in final libraries.

3. Combine the completed reactions and clean with AMPure XP beads (1.5x beads:sample), eluting in 30-40ul.

4. Run cleaned PCR samples on an Agilent Bioanalyzer to quantify molarity and library fragment size distribution. A secondary quantification such as fluorometer (Invitrogen Qubit) or qPCR is also recommended.

5. At this point, samples with distinct multiplexing indices introduced in the PCR can be combined in equimolar ratios to compose a final library for each sequencing lane. It is recommended that you coordinate with your sequencing service provider regarding preferred concentrations and volumes of final libraries.

**Appendix 1: Oligo sequences**

A document [ddRAD oligo table](https://spreadsheets.google.com/spreadsheet/ccc?key=0AnHEwF1NpAxDdEFnWXBEMy02Y2V2UGw4OEJ3SWFtVGc&hl=en_US) is available with sheets detailing sequences for oligos described in this protocol. Worksheets detail oligos for several RE combinations.

NOTES:

Example oligos given here are for EcoRI-MspI ligation as “adapter_P1-EcoRI” and “adapter_P2-MspI”. Both EcoRI and MspI produce 5’ overhangs (AATT and CG respectively). These sequences are therefore present on the phosphorylated 5’ end of the 1.2 and 2.2 oligos. For 3’ overhangs, the appropriate sequences are appended to the 3’ end of 1.1 and 2.1 oligos To switch, for instance, to the 3’ TGCA overhang left by SbfI for the P1 adapter, remove the EcoRI AATT from the 1.2 oligo (for barcode GCATG this becomes /5Phos/CATGCAGATCGGAAGAGCGTCGTGTAGGGAAAGAGTGT) and add the SbfI TGCA to 1.1 (for barcode GCATG this becomes ACACTCTTTCCCTACACGACGCTCTTCCGATCTGCATGTGCA).

An example of this is given for the adapter P1 3’ overhang sequence CATG, referred to as “adapter_P1-flex” because this sequence can be ligated to digests with either SphI (6 base cutter) or NlaIII (4 base cutter). Likewise, an adapter P2 example “adapter_P2-flex” is given that can be ligated to ends produced by either EcoRI (6 base cutter) or MluCI (4 base cutter). Three combinations of these (SphI-EcoRI, SphI-MluCI, and NlaIII-MluCI) permit tuning over 1-2 orders of magnitude in recovered regions genome wide without the need to generate new adapters.

Barcodes are present in both 1.1 and 1.2 oligos as they are part of the double-stranded adapter segment. Barcodes are in reverse-complement in 1.2 oligos. 48 example 5bp barcodes are presented here. Indices are located 5’ of the sequencing primer annealing region of the PCR2 oligos and are in reverse-complement. 12 6bp indices corresponding to the 12 Illumina-supplied multiplexing indices are presented here.

**Appendix 2: Costs (summer 2011)**

Reagents:

EcoR1-HF = 50k Units @ 100K U/mL = 500uL = $169.60

-per sample = $0.35

MSPI = 25K Units @ 20K U/mL = 1.25mL = $185.60

-per sample = $0.02-.05

T4 DNA ligase = 100k Units @ 400k Units/mL = 250uL = $201.60

-per sample = $1.62

Ampure XP beads = 60mL kit = $945.00

-100uL/sample = $1.60

Dynabeads® M-270 Streptavidin = 2mL of beads = $380 (or $1040 for 10mL)

-1uL beads/10ng of DNA (post size-selection) = $0.19

Phusion PCR Kit = 500 amplifications in 20uL = $176.00

3 Rxns/12 samples= .25 rxn/samples = $0.10

Pippin Prep 2% casette = $10/lane = $0.84/sample (if 12 samples pooled/lane of the casette)

Total = $4.56 per sample

Oligo costs: (One-time; we suggest sharing between projects/labs. Standard desalting is sufficient.)

P1 adapters (oligos1.1 and 1.2) = ~$4,000 per 48

P2 adapters (oligos 2.1 and 2.2; each @ 1uM synthesis) = ~$100

PCR primers (for 12) = ~$250

Biotinylated flex-P2.2 oligo (1uM synthesis) = ~$200
