# Supplementary material for: Double Digest RADseq: An Inexpensive Method for De Novo SNP Discovery and Genotyping in Model and Non-Model Species
Source: PLoS One. 2012 May 31;7(5):e37135. doi: 10.1371/journal.pone.0037135 (PMC3365034; doi:10.1371/journal.pone.0037135)
Supplement: Analysis S1 — Additional details on simulations described in this work. Also includes parameter values and run conditions for proof-of-concept analyses in laboratory cross and wild population experiments. (PDF) [file pone.0037135.s003.pdf]

Citation: Peterson, B.K., J.N. Weber, E.H. Kay, H.S. Fisher and H.E. Hoekstra. Double digest RADseq: an inexpensive method for *de novo* SNP discovery and genotyping in model and non-model species.

Supplemental Material

Double digest RADseq: Preliminary expectation

To generate an approximate expectation for our ddRADseq pilot experiments, we created an expected distribution of restriction fragment sizes from *Mus musculus* (Ensembl release 61, NCBI M37) *Rattus norvegicus* (Ensembl release 61, RGSC 3.4) and *Spermophilus tridecemlineatus* (Ensembl release 61, SQUIRREL) by positing a break at each genomic sequence matching the recognition site for either of two restriction enzymes (REs). We retained only those fragments with one of each restriction site, i.e. those generated by double digest (as only these fragments will be sequenced; see protocol). We eliminated "peaks" in this distribution derived from RE sites in high-copy repeats by matching and removing resulting sequences using RepeatMasker 3.2.7 with the "mammal" and "rodentia" repeat databases. From resulting size distributions, we summed counts over proposed size selection windows with mean 200, 300 or 400, and widths of +/- 25bp and +/- 50bp. We used recognition sites for the following REs in pairs: SbfI (8bp site, 75% G/C), SphI (6bp site, 66% G/C), EcoRI (6bp site, 33% G/C), MspI (4bp site, 100% G/C, contains CpG), NlaIII (4bp site, 50% G/C), MluCI (4bp site, 0% G/C). These enzymes were selected because they span the available range of recognition sequence length and G/C composition, and because they all exhibit 100% activity in NEB Buffer 4 (NEB, Ipswitch, MA), allowing all double digest combinations. Resulting fragment size distributions and therefore resulting fragment numbers in each proposed size-selection window were extremely similar across the three rodent genomes; all subsequent analyses used the *Mus* genome.

Double digest RADseq: modeling simulation

We treat the question of region recovery as a function of read depth *d* in a given digest and sizing condition as a series of *d* random draws of from the observed distribution of fragments of each size (fragment size distributions as per "preliminary expectation" above) with probability of recovering a fragment of that size determined by a Normal sampling distribution of mean equal to our size selection mean and standard deviation *s*. To obtain *s*, the actual standard deviation of our size sampling distribution in our experimental data, we compared simulations to real data derived from individuals of several species of deer mouse (genus *Peromyscus,* which diverged from *Mus* approximately 25 Mya [1] representing approximately 40% divergence in non-coding sequence [data not shown]). Sequence data were generated using EcoRI - MspI digests, and were size selected either by "narrow" Pippin Prep selection (set at 300 ± 24bp), a "wide" Pippin Prep selection (set at 300 ± 36bp), or agarose gel slab excision at 300bp of approximately 50-100bp total width. Simulations were run over a range of total read counts matched to real data and with *s* from 1 to 100 (figure S1). We observed very reproducible best fits to both Pippin Prep conditions across pools with average *s* = 11.5bp (r2 0.99) for the "narrow" sizing and *s* = 17.5bp (r2 0.98) for the "wide" sizing. Gel excision best-fit *s* values varied from 20-40bp (r2 0.94), but were not reproducible across pools.

Because reduced representation libraries are expected to consist of primarily fragments derived from the peak of size selection efficiency along with less prevalent random inclusion of fragments at increasing size deviation from the sizing mean, region recovery and read coverage is expected to be best correlated between individuals and experiments for regions coming from peak size selection efficiency. Therefore, while both real and simulated data show that per-individual number of regions recovered is a function of read depth and continues to increase well beyond the total read counts sampled in our experiments, the average number of shared regions saturates quickly (main text figure 4, panels C, D). While in part this is due to poorly covered individuals reducing the calculated mean of shared regions for all individuals, mean shared region counts computed only on the most highly sampled data points (>200,000 reads in “narrow” Pippin Prep and >400,000 reads in “wide” Pippin Prep; figure S2) continue to show this effect of saturation of shared regions.

Furthermore, mean shared region counts in these “saturated-only” subsets (figure S2) correspond well with the transition between exponential and logarithmic accumulation of new regions with additional sequencing investment seen in individual region counts in both simulation and real data (figures 4C, S3). This is consistent with the hypothesis that this transition corresponds to the saturation of regions lying between restriction sites at spacings well recovered by the size selection condition and the slow addition of randomly recovered fragments from the tails of the size sampling distribution (and which are not expected to be shared between individuals). Furthermore, the observation that samples sized by gel excision do not appear to display the same saturation kinetics suggests that this saturation is a function of differential precision in size selection in the two methods.

Saturation is characterized in both real and simulated data by the point at which the rate of change in recovered regions with additional sequencing ceases to drop (coming off of the peak rate of change at the switch from logistic to asymptotic increase), and we therefore report this value (convergence of the second derivative at zero; figure S3) in both the X axis (sequencing investment) and Y axis (recovered region count) in table 1 for a variety of source genomes and restriction enzyme pairs.

Random shearing RADseq: simulation

To compare expected recovery of regions at various read depths between double RE digest and random shearing RADseq approaches, we sought a pair of conditions that are expected to yield the same final genotyping depth (7x) sequence regions counts at approximately the same final target depth at saturation (as defined above and in figure S3) for ddRADseq, and as follows for random shearing RADseq. As most published work employing RADseq to date has used the SbfI restriction enzyme, we began by simulating recovery randomly from a number of fragments equal to twice the number of SbfI sites in the *Mus musculus* genome. We assume that the restriction digest is complete (i.e. all recognition sites for the enzyme are cut) and that shearing is random, and therefore that each SbfI recognition site in the genome produces two sequencing library ends (one in each direction from the cut site). A cut site is considered to be “recovered” from a simulated sample if either resulting end is sampled at or above 7x. As the other fragment resulting from that cut site is functionally redundant, each site is only considered

to contribute one region. The analysis summarized in figure S4 shows the number of regions expected to be recovered at or above 7x coverage from 20 or greater of 24 simulated individuals.

For these simulations, random shearing RADseq showed the expected sharply cooperative transition (i.e. steep slope) from zero regions shared between intervals, to complete recovery of at least one region adjacent to each cut site in all or nearly all individuals. As 60,000 cuts generating 120,000 fragments require at least 840,000 reads perfectly evenly distributed to achieve 7x, an observed transition from essentially no shared region recovery to complete saturation between 800,000 and 1,400,000 reads in random sampling simulations is consistent with expectation. When scaled for genome size this is roughly the value reported in [2], in which Cutthroat Trout with a genome 75% the size of the *Mus* genome saw approximately 50% (~19,000 of ~40,000) of regions shared in 20 or more of 24 individuals at an average of ~830,000 processed reads per individual, which in our simulations with *Mus* occurred at 1,100,000 reads (corrected for genome size, this would correspond to 825,000 reads in Cutthroat Trout).

Region recovery: ddRADseq vs. random shearing

A RADseq library constructed with SbfI and random shearing (following [3]) from *Mus musculus* is expected to saturate at 1.3M reads, with 60,200 unique regions in 20 of 24 simulated individuals (see above). To compare recovery as a function of read investment between our double RE digest approach and random shearing, we employed our “wide” size-selection-trained sampling model to a variety of double digest fragment distributions from the *Mus musculus* genome in search of a similar result. From these simulations we chose a ddRADseq experiment with SphI and MluCI and selecting 300bp ± 36bp. Simulations suggest this will saturate at 1.3-1.4M reads and yield 60,600 unique regions in 20 of 24 simulated individuals. While similar in coverage and shared region counts at saturation, the ddRADseq simulation is substantially more robust to fluctuations in coverage across individuals, yielding an expected ~35,000 well-covered regions (58% of the set expected at saturation) shared across individuals at 700K total reads per individual (50% the expected read count for saturation). In contrast, the RADseq simulation suggests that fewer than 100 regions are expected to reach the 7x coverage required for genotype inference at 700K reads. While the RADseq simulations climb to 19,000 (32% of final) expected shared regions at 1M reads (75% of saturation), at this read count ddRADseq is expected to have recovered nearly 50,000 (83% of total) shared regions (Figure S4). These properties of robustness to under-sampling and predictability of saturating read counts from simulation data permit design and execution of genotyping experiments with little waste. In our preliminary results with EcoRI and MspI in *Peromyscus*, the estimate of 300,000 reads required to confidently sample 20,000-30,000 regions per individual appears accurate, but even those individual samples receiving fewer than half of the reads required to saturate recovery still shared thousands of high-coverage regions (Figure 4D, main text).

Library Construction Summary

ddRADseq library construction consists of five steps: annealing complementary oligonucleotides to form barcoded adapters P1 and P2, digesting genomic DNA with two REs, ligating P1 and P2

barcoded adapters onto the ends of digested fragments, size selecting from the ligation products, and PCR amplifying the remaining subset of fragments. To create adapters, we first annealed two sets of complementary, single-stranded oligos (Integrated DNA Technologies) in equimolar ratios in 1x annealing solution (see protocol). Annealing was accomplished by increased the temperature of this mixture to 97.5-C for 150 seconds, and then slowly cooling the mixture to room temperature. Genomic DNA for the RE digests was extracted either with an affinity column (DNeasy Kit, Qiagen, Valencia, CA) or through phenol-chloroform precipitation (performed using an Autogenprep 965 [Autogen, Inc., Holliston, MA] automated extraction machine), and all samples were RNAse treated. We performed each digestion at 37C for 3 hours without a heat kill (hold at 4°C), and included 0.5-1ug genomic DNA quantified by fluorometry using Quant-It dye, (Invitrogen, Grand Island, NY), 2 Units EcoRI-HF, 40-100 Units MspI, 10X NEB Buffer 4 (NEB, Ipswitch, MA) and H_2O, up to a total volume of 20-30ul (or 50ul reactions for double RE digests with 5ug input DNA). After the digests, we cleaned each sample with Agencourt AMPure XP magnetic beads (Beckman Coulter Genomics, Danvers, MA). For ligations, we combined 50-500ng of digested DNA based on pre-ligation concentrations, a 10-fold excesses of adapters relative to the expected number of complementary restriction overhangs in the genomic fragments, and 20 Units of T4 DNA ligase and ligase buffer (NEB). We then added water up to a total volume of 40ul, incubated the reaction at 37°C for 30 min, and heat killed the ligase by incubating at 65°C for 10 min, followed by a slow cooling to room temperature. After ligations, samples were again cleaned with Agencourt AMPureXP beads. Next, we pooled samples and size-selected fragments by gel excision or by Pippin Prep (Sage Science, Beverly, MA). We then amplified size-selected fragments using a Phusion High-Fidelity PCR Kit (Finnzymes) for 10 cycles. We performed multiple amplification reactions for each size-selected sample, each with 3-4ul template. We then pooled reactions, performed an Agencourt AMPureXP bead clean-up, and measured final concentrations on a Bioanalyzer (Agilent, Santa Clara, CA).

These libraries do differ from standard Illumina libraries in one respect that bears consideration: the Illumina platform requires that the in-line barcodes (i.e. adapter barcodes) pooled for an experiment be base-composition-balanced at each position. For example, barcodes ACGT, TACG, GTAC, CGTA make a suitable pool because each base position in the pool has all four nucleotides in equal quantities (assuming pooling of equivalent molar quantities of each barcode), By contrast, although barcodes ACGT, ATCG, AGTC, AGCT are unique sequences, this pool is invalid as only Adenine is present in base position one. Thus, when composing pools of barcodes to combine, it is important to use sets of barcodes that yield balanced compositions of all four nucleotides in each sequence position.

Genotyping in a laboratory generated cross: Linkage map construction

The following procedures were all conducted using the R/qtl software package [4] to generate a linkage map for a genetic cross between *P. maniculatus* and *P. polionotus* (strains as described in [5]). First, we imported genotypes from 192 backcross offspring that were either homozygous for *P. maniculatus* allele or heterozygous, having both *P. maniculatus* and *P. polionotus* alleles. We excluded homozygous *P. polionotus* genotypes because, given our cross design, these are probably located on the *P. polionotus* Y-chromosome, influenced by segregation distortion or

are erroneous genotypes (only a small fraction of all genotypes were homozygous for *P. polionotus* alleles = 2,131/316,412). Second, we estimated the pairwise recombination frequencies between all pairs of markers using the “est.rf” function. Third, we organized autosomal markers into linkage groups with the “formLinkageGroups” function. Setting the min.rf and max.lod parameters to 0.08 and 9, respectively, separated all markers into 60 linkage groups, 25 of which contained more than 5 markers. Since remaining groups were composed primarily of markers with many missing genotypes, we retained only the largest 25 linkage groups for further analyses. Finally, after examining by eye the recombination frequencies and LOD scores for linkage between all markers, we were able to confidently collapse our dataset into 24 total linkage groups, which is consistent with the haploid karyotype both *P. maniculatus* and *P. polionotus*.

To determine the order of markers within each linkage group, we initially used the “orderMarkers” function. This function uses a computationally fast algorithm to minimize the number of crossovers (or maximize the likelihood) of marker order within a sliding window. Using a window size of 6 markers provided a good order for most linkage groups. However, the “orderMarkers” function is biased toward examining markers with the most complete genotype information first. This bias can create blocks of incorrectly ordered markers when data sets have either large numbers of markers or the chosen window size is not sufficiently wide. We identified, by eye, several regions with suspect orders and manually moved these markers. We then used the ripple function to minimize the number of crossovers on the modified linkage groups. These modifications resulted in a set of well-ordered linkage groups, with a minimized number of total crossovers per chromosome and low frequencies of recombination between adjacent markers) (figure 5A,B). Finally, we estimated the genetic distances between all markers using the est.map() function. Analyzing all 1,110 remaining markers with the kosambi map function produced a densely covered, sex-averaged map with a total length of 1,759.7cM and an average intermarker distance of 1.6cM (figure 5B). The total length of our map is comparable to other rodents with known genetic map lengths, such as *Mus* and *Rattus* [6].

Genotyping in a wild population: *Peromyscus leucopus* sampling
We collected 92 *P. leucopus* DNA samples from four states: (1) Tensas Parish, Louisiana (326' 16.260" N, 91°29'50.640" W; N=55), (2) La Salle Parish, Louisiana (31°29'42.000" N, 92°0'42.180" W; N=16) (2) Cherry Co., Nebraska (Site 1: 42°53'25.920" N, 100°31'5.460" W, N=1; 1 voucher specimen MCZ66476; Site 2: 42°51'5.640" N, 100°31'14.280" W; N=5; 5 voucher specimen MCZ66485, MCZ66491, MCZ66494, MCZ66497, MCZ66502; Site 3: 42°42'42.360" N, 100°37'6.300" W, N = 1; 1 voucher specimen MCZ66607), (3) Westmoreland Co., Pennsylvania (40°8'45.600" N, 79°16'8.400" W; N=3), and (4) Middlesex Co., Massachusetts (42°22'11.34" N, 71°6'25.18" W; N=4; 2 voucher specimen MCZ63293-63294). In addition, *P. leucopus* originally derived from the Peromyscus Genetic Stock Center colony (N=5,collected near Linville, North Carolina) were included in our analysis. We generated an allele frequency spectrum from the Tensas Parish, Louisiana population (figure 5C). A genetic principal component analysis was run with the remaining wild-caught and lab-reared individuals (figure 5D).

Literature Cited:

1. Ramsdell CM, Lewandowski AA, Glenn JLW, Vrana PB, O'Neill RJ, et al. (2008) Comparative genome mapping of the deer mouse (*Peromyscus maniculatus*) reveals greater similarity to rat (*Rattus norvegicus*) than to the lab mouse (*Mus musculus*). BMC Evolutionary Biology 8: 65. doi:10.1186/1471-2148-8-65.

2. Hohenlohe PA, Amish SJ, Catchen JM, Allendorf FW, Luikart G (2011) Next-generation RAD sequencing identifies thousands of SNPs for assessing hybridization between rainbow and westslope cutthroat trout. Molecular Ecology Resources 11: 117–122. doi:10.1111/j.1755-0998.2010.02967.x.

3. Baird NA, Etter PD, Atwood TS, Currey MC, Shiver AL, et al. (2008) Rapid SNP discovery and genetic mapping using sequenced RAD markers. PLoS One 3: e3376. doi:10.1371/journal.pone.0003376.

4. Broman KW, Wu H, Sen S, Churchill GA (2003) R/qtl: QTL mapping in experimental crosses. Bioinformatics 19: 889–890. doi:10.1093/bioinformatics/btg112.

5. Weber JN, Hoekstra HE (2009) The evolution of burrowing behaviour in deer mice (genus *Peromyscus*). Animal Behaviour 77: 603–609. doi:10.1016/j.anbehav.2008.10.031.

6. Dietrich WF, Miller J, Steen R, Merchant MA, Damron-Boles D, et al. (1996) A comprehensive genetic map of the mouse genome. Nature 380: 149–152. doi:10.1038/380149a0.

Flowcell	Lane	Run Type	Multiplex Index	Size Selection Pool	Pool Reads	Sample Count	Mean Reads Per Sample	Lane Totals	
100617	8	PE	None	HFparents	15800310	8	1975038.8		
100617	8	PE	None	1	2557405	3	852468.3	**Total Reads**	23,811,226
100617	8	PE	None	3	2289092	6	381515.3	**Assigned Reads**	22,944,902
100617	8	PE	None	2	2298095	6	383015.8	**Assignment Rate**	96%
100507	3	SR	None	1	3922069	8	490258.6		
100507	3	SR	None	3	3590076	8	448759.5		
100507	3	SR	None	2	3152646	8	394080.8		
100507	3	SR	None	5	3085189	8	385648.6	**Total Reads**	19,649,924
100507	3	SR	None	4	2967611	8	370951.4	**Assigned Reads**	19,475,849
100507	3	SR	None	6	2758258	8	344782.2	**Assignment Rate**	99%
101013	7	SR	None	1	4542792	12	378566		
101013	7	SR	None	3	4169598	12	347466.5	**Total Reads**	19,777,269
101013	7	SR	None	2	5886668	12	490555.7	**Assigned Reads**	19,671,663
101013	7	SR	None	4	5072605	12	422717.1	**Assignment Rate**	99%
101029	7	SR	None	1	3284793	11	298617.5		
101029	7	SR	None	3	3239037	12	269919.8	**Total Reads**	25,631,225
101029	7	SR	None	2	9194077	13	707236.7	**Assigned Reads**	25,437,980
101029	7	SR	None	4	9720073	12	810006.1	**Assignment Rate**	99%
110927	1	SR	1	1	12965322	12	1080443.5		
110927	1	SR	1	3	14739468	12	1228289		
110927	1	SR	1	2	13640244	12	1136687		
110927	1	SR	1	4	14485373	12	1207114.4		
110927	1	SR	5	EK5	59314298	48	1235714.5	**Total Reads**	233939689
110927	1	SR	6	EK6	56940414	48	1186258.6	**Assigned Reads**	221,189,445
110927	1	SR	7	EK7	49104326	48	1023006.8	**Assignment Rate**	95%

Supplemental Table 1. Read Counts by Lane, Pool and Index. Reads generated are reported by size selection pool (3 – 48 barcoded individual samples each; see Sample Count). Run type indicates paired-end or single-read sequencing. Where applicable, Multiplex index indicates the standard Illumina multiplexing read index used. Pool reads indicates the number of reads uniquely assigned to individuals in each pool (by inline barcode and multiplexing index as indicated; see “Sample multiplexing via combinatorial indexing” in methods). Total number of reads for each lane, total number of reads uniquely assigned to individual samples, and rate of assignment success are summarized under Lane Totals.

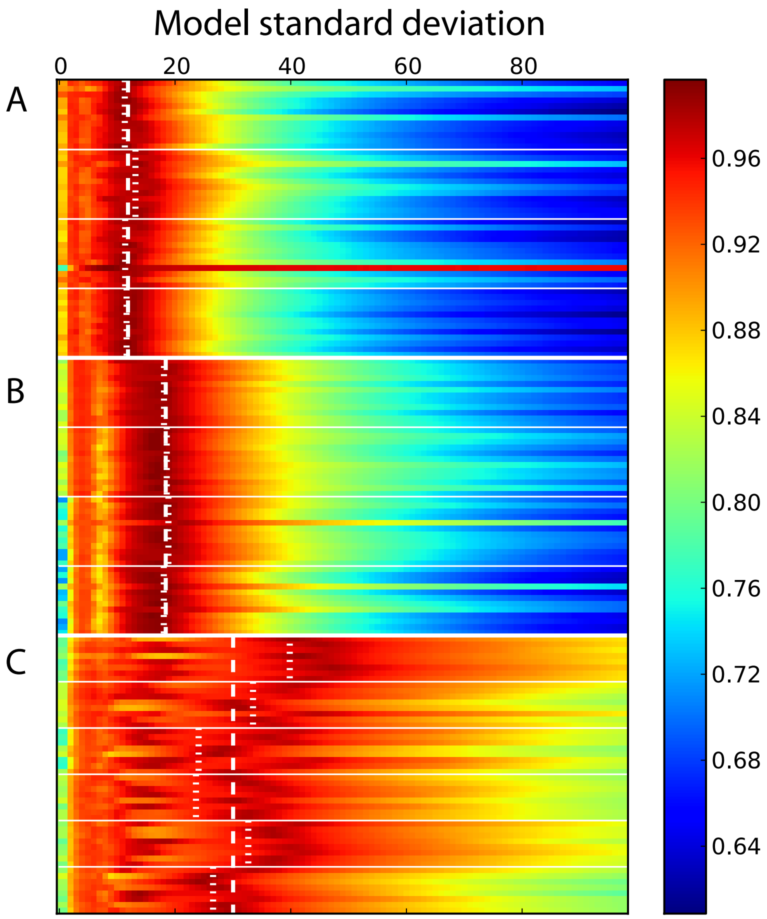

Supplemental Figure 1. Goodness-of-fit of simulation to real data across sampling distribution standard deviations illustrates precise and repeatable size selection using Pippin Prep automated size selection. Horizontal lines in the heatmap correspond to goodness-of-fit (Pearson's R^2 as indicated in legend, right) of a normal sampling with mean=300bp and SD indicated by heatmap column (from 1 to 100bp, left to right) for each of 144 individuals (one heatmap line per individual) to the observed ranked region coverage. (A) Fragments generated by size selection via Pippin Prep (size range 300bp +/-24) and (B) independent replicate sample using the same size selection mean (300bp) with 50% broader size range (+/-36bp) and sequenced in the same lane. (C) Fragments generated by gel extraction, also sequenced in the same flowcell lane. Vertical dotted lines indicate the average (weighted by total reads per individual) of best-fit standard deviations for individuals in a single size selection cohort (Pippin Prep channel or gel lane) and Vertical dashed lines indicate weighted average (as above) of best-fit standard deviations for all individuals in a single flowcell lane (i.e. all 48 individuals subject to that size selection regime, across cohorts).

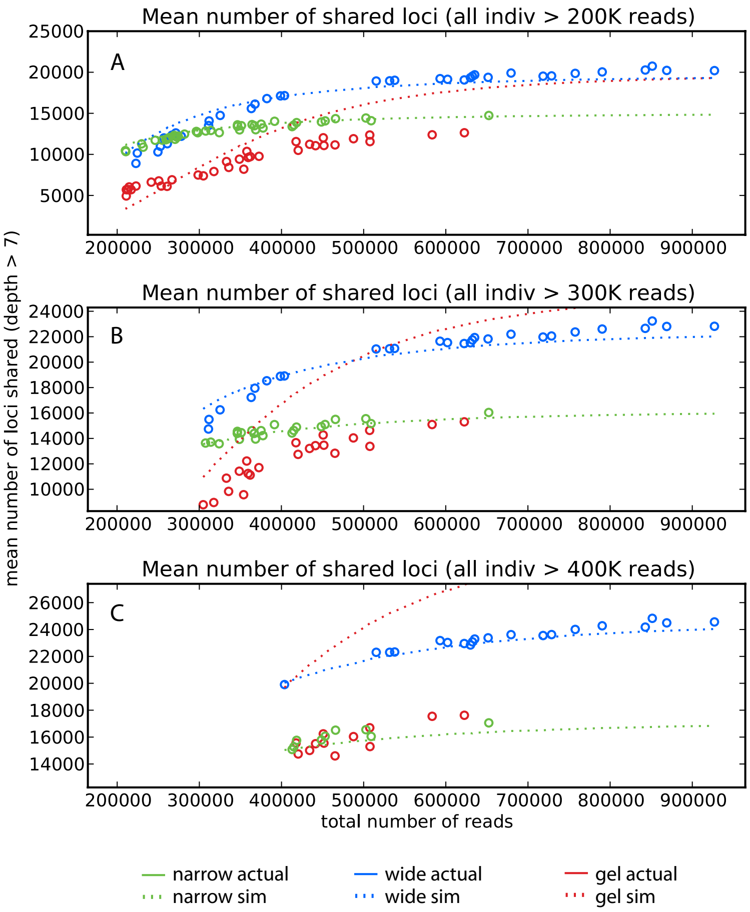

Supplemental Figure 2. Restricting shared region calculation to the most highly sampled individuals in real and simulated data demonstrates that shared region saturation is reached in Pippin Prep conditions. Recomputed mean number of shared loci across all individuals (see figure 4D), excluding all individuals below indicated total read count threshold for each panel with sequence read cutoffs of (A) >200kb, (B) >300kb and (C) >400kb. As in figure 4D, dashed lines are simulated data (see main text; "model simulation" in supplementary text) and solid circles are observed data for a single individual. While both Pippin Prep conditions show no increase in average read count after excluding individuals below saturation (>200-300K reads for "narrow", >300-400K reads for "wide"), mean shared region values for high read-count gel extraction samples show no evidence of saturation with removal of low read-count individuals (peak means = (A) 12K, (B)15K, and (C) 18K.

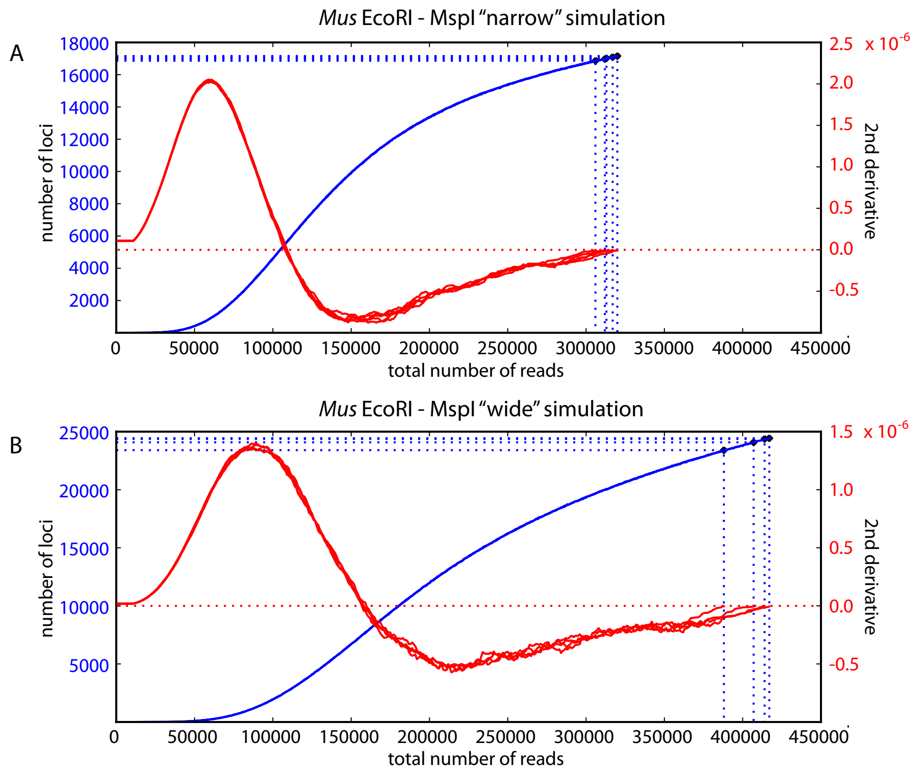

Supplemental Figure 3. Recovery saturation in simulations from *Mus* predicts shared region saturation in experimental data from *Peromyscus*. Blue lines indicate number of loci expected in (A) "narrow" and (B) "wide" automated size selection simulation conditions (see text; "model simulation" supplementary text) as a function of number of total reads for 5 example simulation runs (left Y-axis). Red lines show second derivatives of smoothed values for these simulations (right Y-axis; red dashed line is 0 in second derivative). Simulations are labeled "saturated" for reads at the transition from logistic to asymptotic recovery as the second derivative goes to zero. Consistent with the hypothesis that shared regions in multiple individuals are largely comprised of regions recovered at high efficiency (logistic recovery), this single-sample simulation recovery saturation of ~17,000 regions for "narrow" and ~24,000 regions for "wide" size selection is highly concordant with observed saturation in shared region recovery in observed data at or above this saturation in read count (figures 4D; S2).

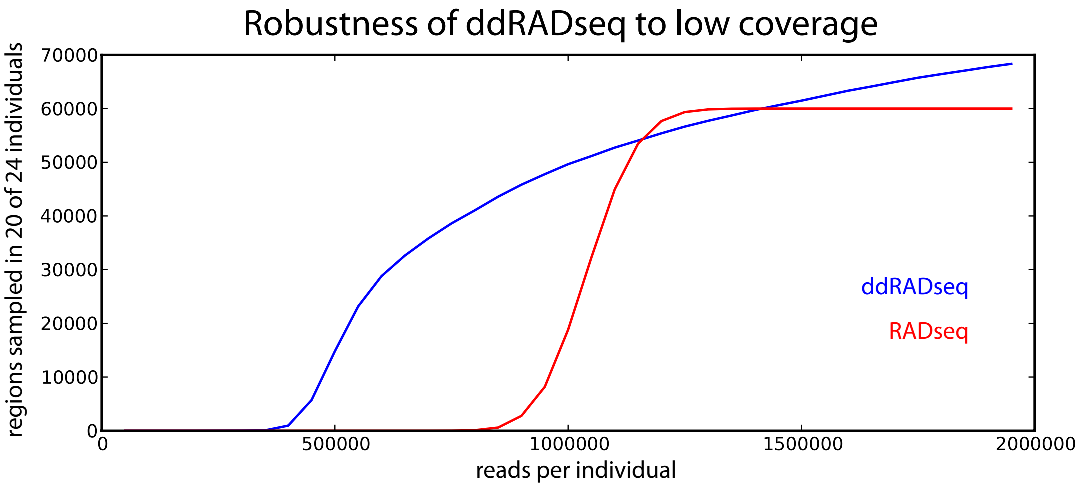

Supplemental Figure 4. A ddRADseq experiment targeting equivalent numbers of loci at saturation displays more robust shared region recovery at low read counts. Red line indicates number of regions recovered at genotyping depth (7x or higher) in 20 or more of 24 individual simulations of random shearing RADseq [3] performed on the *Mus musculus* genome using the RE SbfI. Blue line indicates the number of regions recovered at genotyping depth (7x or higher) in 20 out of 24 ddRADseq simulated individuals for a mean=300bp, SD=20bp size selection using a SphI - MluCI digest of the *Mus musculus* genome (see text). Due to correlated read counts for regions between individual samples (see "Method Overview", main text), ddRADseq begins to recover significant numbers of high-coverage shared regions between individuals at substantially lower total read counts.
